# Pitfalls of Single Measurement Screening for Diabetes and Hypertension in Community-Based Settings

**DOI:** 10.5334/gh.1083

**Published:** 2021-12-03

**Authors:** Stephen Olivier, Thomas Murray, Philippa Matthews, Ngcebo Mhlongo, Resign Gunda, Kathy Baisley, Dickman Gareta, Tshwaraganang Modise, Theresa Smit, Kobus Herbst, Xolile Mpofana, Thumbi Ndung’u, Olivier Koole, Deenan Pillay, Willem Hanekom, Emily Wong, Mark J. Siedner, Vukuzazi Study team

**Affiliations:** 1 Africa Health Research Institute, KwaZulu-Natal, ZA; 2 Islington GP Federation, London, UK; 3 London School of Hygiene and Tropical Medicine, London, UK; 4 DSI-MRC South African Population Research Infrastructure Network (SAPRIN), Durban, ZA; 5 Division of Infectious Diseases, University of Alabama Birmingham, Birmingham, AL, US; 6 Harvard Medical School, Boston, MA, US; 7 HIV Pathogenesis Programme, The Doris Duke Medical Research Institute, Nelson R. Mandela School of Medicine, University of KwaZulu-Natal, ZA; 8 Max Planck Institute for Infection Biology, Berlin, DE; 9 Division of Infection and Immunity, University College London, London, UK

**Keywords:** hypertension, Diabetes, Screening

## Abstract

**Background:**

Cross-sectional screening programs are used to detect and refer individuals with non-communicable diseases to healthcare services. We evaluated the positive predictive value of cross-sectional measurements for Diabetes Mellitus (DM) and hypertension (HTN) as part of a community-based disease screening study, ‘Vukuzazi’ in rural South Africa.

**Methods:**

We conducted community-based screening for HTN and DM using the World Health Organization STEPS protocol and glycated haemoglobin A1c (HbA1c) testing, respectively. Nurses conducted follow-up home visits for confirmatory diagnostic testing among individuals with a screening BP above 140/90 mmHg and/or HbA1c above 6.5% at the initial screen, and without a prior diagnosis. We assessed the positive predictive value of the initial screening, compared to the follow up measure. We also sought to identify a screening threshold for HTN and DM with greater than 90% positive predictive value.

**Results:**

Of 18,027 participants enrolled, 10.2% (1,831) had a screening BP over 140/90 mmHg. Of those without a prior diagnosis, 871 (47.6%) received follow-up measurements. Only 51.2% (451) of those with completed follow-up measurements had a repeat BP>140/90 mmHg at the home visit and were referred to care. To achieve a 90% correct referral rate, a systolic BP threshold of 192 was needed at first screening. For DM screening, 1,615 (9.0%) individuals had an HbA1c > 6.5%, and of those without a prior diagnosis, 1,151 (71.2%) received a follow-up blood glucose. Of these, only 34.1% (395) met criteria for referral for DM. To ensure a 90% positive predictive value i.e. a screening HbA1c of >16.6% was needed.

**Conclusions:**

A second home-based screening visit to confirm a diagnosis of DM and HTN reduced health system referrals by 48% and 66%, respectively. Two-day screening programmes for DM and HTN screening might save individual and healthcare resources and should be evaluated carefully in future cost effectiveness evaluations.

## Introduction

Type 2 diabetes mellitus (DM) and hypertension (HTN) are increasing in prevalence worldwide and are major contributors to the rise in the global burden of non-communicable diseases (NCDs) [1]. It is estimated that raised systolic blood pressure caused 10.4 million deaths worldwide in 2017 and was the leading risk factor for all-cause mortality [2]. Similarly, the number of people living with DM is expected to increase to 700 million in 2045 from 463 million in 2019 [3]. Low- and middle-income countries are shouldering an increasing burden of DM and HTN morbidity and mortality [4]. For example, the recent South African demographic health survey estimated that 13% of women and 8% of men had diabetes and 46% of women and 44% percent of men suffered from hypertension [5]. Moreover, that survey found that approximately 70% of people with either hypertension or diabetes were unaware of their diagnosis. Consequently, after tuberculosis, DM and cerebrovascular disease are now the second and third-leading causes of death in the country [6].

In response to the growing burden of undiagnosed DM and HTN in South Africa, there are increased efforts to enhance disease detection through screening. This includes routine clinic-based screening for NCDs [7], but also community-based or community healthcare worker-led screening for those not captured by the formal healthcare system [8,9,10,11,12,13,14]. Such initiatives are often implemented using cross-sectional screening followed by referral to local primary healthcare clinic services for individuals with abnormal results. However, the impact on both patients and health services of referring people based on a single measurement is not well established. Such programs must weigh the benefits of establishing care for those previously undiagnosed against overburdening the healthcare system with unnecessary referral of individuals without established disease, and the additional costs and burden to individuals who may also be inconvenienced or exposed to nosocomial infection by avoidable clinic visits.

## Methods

### Study Setting

From May 2018 to Dec 2019, the Africa Health Research Institute (AHRI) conducted a community-based survey, named ‘Vukuzazi’ that screened individuals for infectious (HIV and TB) and non-communicable diseases (hypertension and diabetes) [15] in its Demographic and Health Surveillance (DHS) catchment area located in the uMkhanyakude district of KwaZulu-Natal across an area of approximately 850 km^2^. The area is one of the lowest ranked in terms of health and socioeconomic status and is catered to by 11 government-run public health clinics [16,17]. A central element of the study was referral of participants with new or uncontrolled disease to local clinics for establishment or re-engagement in care. To prevent overburdening the local health system with potentially unnecessary referrals, the program included a second, home-based visit by a study nurse to confirm diagnosis of DM and HTN in those who screened positive at the initial health fair. In this analysis, we aimed to assess the positive predictive value of a single screening measurement for DM and HTN compared to sequential testing with repeated measures across two separate days. We hypothesized that a significant proportion of individuals who would have been referred based on measures from a single day would no longer meet the criteria for referral during their second visit. Our over-arching aim was to assess the value of repeated measures of NCD screening prior to health system referrals in rural South Africa.

### Study population

All residents aged 15 years and above in the DHS were eligible for participation. Study staff visited all households in the surveillance area and invited eligible household members to attend a mobile health screening fair, which traversed the surveillance area over the course of an 18-month period to conduct study procedures.

### Study Measures

Study staff administered standardized questionnaires to collect current and past medical history, including participation in care and medication use for DM and HTN during the health screening fair. Blood pressure was recorded using Omron portable electronic sphygmomanometer (Omron Global, Kyoto Japan) using the World Health Organization STEPs protocol [18], three measures on the same upper-arm in the seated position were done, with the first measure being done after the participant had been seated for 15 minutes, during which time the questionnaire was administered. The subsequent second and third measurements were then taken with three minutes between each reading. When the standard cuff size (22–32 cm) was too small a large cuff size (33–43 cm) was used. Whole blood was collected into ethylenediaminetetraacetic acid (EDTA) tubes for measurement of glycated haemoglobin (HbA1c, VARIANT II TURBO, Bio-Rad, Marnes-la-Coquette, France).

### Study Definitions

We calculated BP using the average of the last two of three measurements collected. Elevated BP was defined as a systolic BP ≥140 mm Hg and/or diastolic BP ≥90 mm Hg, based on South African Department of Health Primary Care Guidelines [19]. For participants with systolic BP ≥180mmHg or diastolic BP ≥110mmHg at the initial primary health fair, symptoms of hypertensive emergency were assessed, and if present referral for urgent clinical care was arranged. A study nurse conducted a home visit for confirmatory testing in those with elevated blood pressure at the health fair, who were not currently on treatment. If the blood pressure was again elevated at the home visit, the participant was referred to the local primary healthcare clinic for management of hypertension. All participants were given lifestyle advice by the study nurse.

Individuals with an HbA1c > 6.5% at the health fair and not currently on diabetes treatment were also seen at their homes by a study nurse for a point-of-care blood glucose measurement. Blood glucose testing using a portable capillary sample was used in place of HbA1c for the home-based confirmation measurement because it is the standard of care for diabetes diagnosis in South Africa [20]. Participants were asked to fast before the follow up measure but only a minority of participants reported doing so. If blood glucose was raised above 7.0 mmol/L (fasting) or above 11.0 mmol/L (non-fasting) the participant was referred to a local primary healthcare clinic for management of diabetes.

### Statistical Analysis

For this analysis, we included individuals attending the Vukuzazi health fair who 1) screened positive for elevated BP or elevated HbA1c at the health fair and did not report being on therapy for HTN or DM, respectively; and 2) were visited at home by a study nurse for confirmatory disease measurement. Study analytic samples were divided it into sub-samples of those with elevated BP and those with elevated HbA1c at the health fair. We graphically depicted and correlated initial and confirmatory systolic and diastolic BP measurements and glucose tolerance measurements between the health fair and confirmatory visits. We then estimated the positive predictive value of the initial screen for elevated BP (elevated diastolic or systolic) and HbA1c measures by comparing them to a reference standard defined as sequentially positive screens at both the health fair and at the home visit). We then fitted a logistic regression model with a positive sequential screen at both the health fair and the home visit as the outcome of interest, this allowed us to predict marginal probabilities and to estimate how changes in BP and HbA1c at the screening visit increased the probability of a confirmed diagnosis after two visits. Finally, we determined the threshold of initial screening BP and HbA1c that would result in >90% of those being referred as having a confirmed disease after a second measure. In a sensitivity analysis, we excluded individuals from the DM sample with a confirmatory non-fasting blood glucose between 7.0–11.0 mmol/L, who might have a diagnosis of diabetes that cannot be determined by their non-fasting status. All data analysis was conducted using R version 4.0.2 [21].

### Ethics Statement

The Vukuzazi Study protocol was reviewed and approved by the Biomedical Research Ethics Committee at the University of KwaZulu-Natal (BE560/17), by the Ethics Committee of the London School of Hygiene (#14722) and by the Partners Institutional Review Board (2018P001802). All study participants gave written informed consent.

## Results

### Study Sample

**Figure 1:**
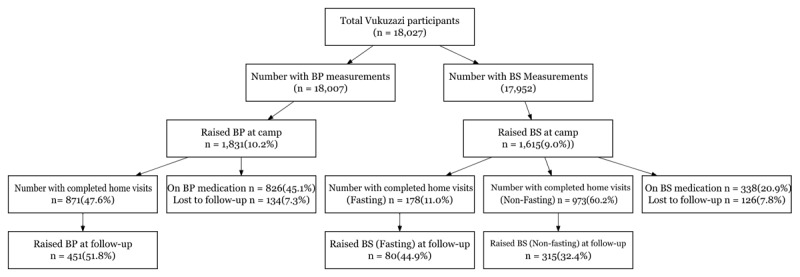
Flow diagram of participants included in the analysis with initial and repeated elevated measures of blood pressure and HbA1c.

Of the 18,007 participants who had their BP measured at the Vukuzazi health fair, 1,831 (10.2%) had high BP defined as either a systolic BP at least 140 mmHg or diastolic BP over 90mmHg (Figure 1). Of these 45.1% already carried a diagnosis of HTN and were referred to clinic for optimization of treatment, 7.3% were unable to be recontacted/LTFU. 871 (47.6%) completed their second screening visit at home with a study nurse and comprised our elevated BP study sample. Of the17,952 participants who completed HbA1c testing at the Vukuzazi fair, 1,615 (9%) had a HbA1c > 6.5% (Figure 1). Of these 20.9% already carried a diagnosis of DM and were referred to clinic for optimization of treatment, 7.8% were unable to be recontacted/LTFU. One thousand one hundred and fifty-one (71.3%) had follow-up blood glucose measurements, of which 178 (11.0%) were fasting and 973 (60.2%) non-fasting measures. Participant characteristics with elevated BP and HbA1c at the health fair are shown in Table 1 compared to the overall population. Supplementary Table 1 shows the characteristics of those who completed home visits compared to those LTFU.

**Table 1:** Cohort Demographics.

	Elevated blood Pressure	Elevated blood sugar	Overall population

Characteristic	N = 871^1^	N = 1,151^1^	N = 18,027^1^
**Age**	58 (45, 68)	60 (51, 68)	37 (23, 56)
**Age categories**			
15–24	36 (4.1%)	26 (2.3%)	4,967 (28%)
25–44	172 (20%)	123 (11%)	6,000 (33%)
45–64	374 (43%)	628 (55%)	4,593 (25%)
65+	289 (33%)	374 (32%)	2,467 (14%)
**Sex**			
Male	277 (32%)	196 (17%)	5,800 (32%)
Female	594 (68%)	955 (83%)	12,227 (68%)
**BMI**	30 (25, 36)	33 (28, 38)	26 (22, 32)
**Systolic BP**	147 (141, 159)	125 (114, 138)	114 (103, 125)
**Diastolic BP**	91 (82, 97)	76 (68, 83)	70 (63, 78)
**HbA1c %**	5.80 (5.50, 6.20)	7.10 (6.60, 10.20)	5.70 (5.40, 6.00)

^1^ Median (IQR); n (%).

### Hypertension Screening

**Figure 2:**
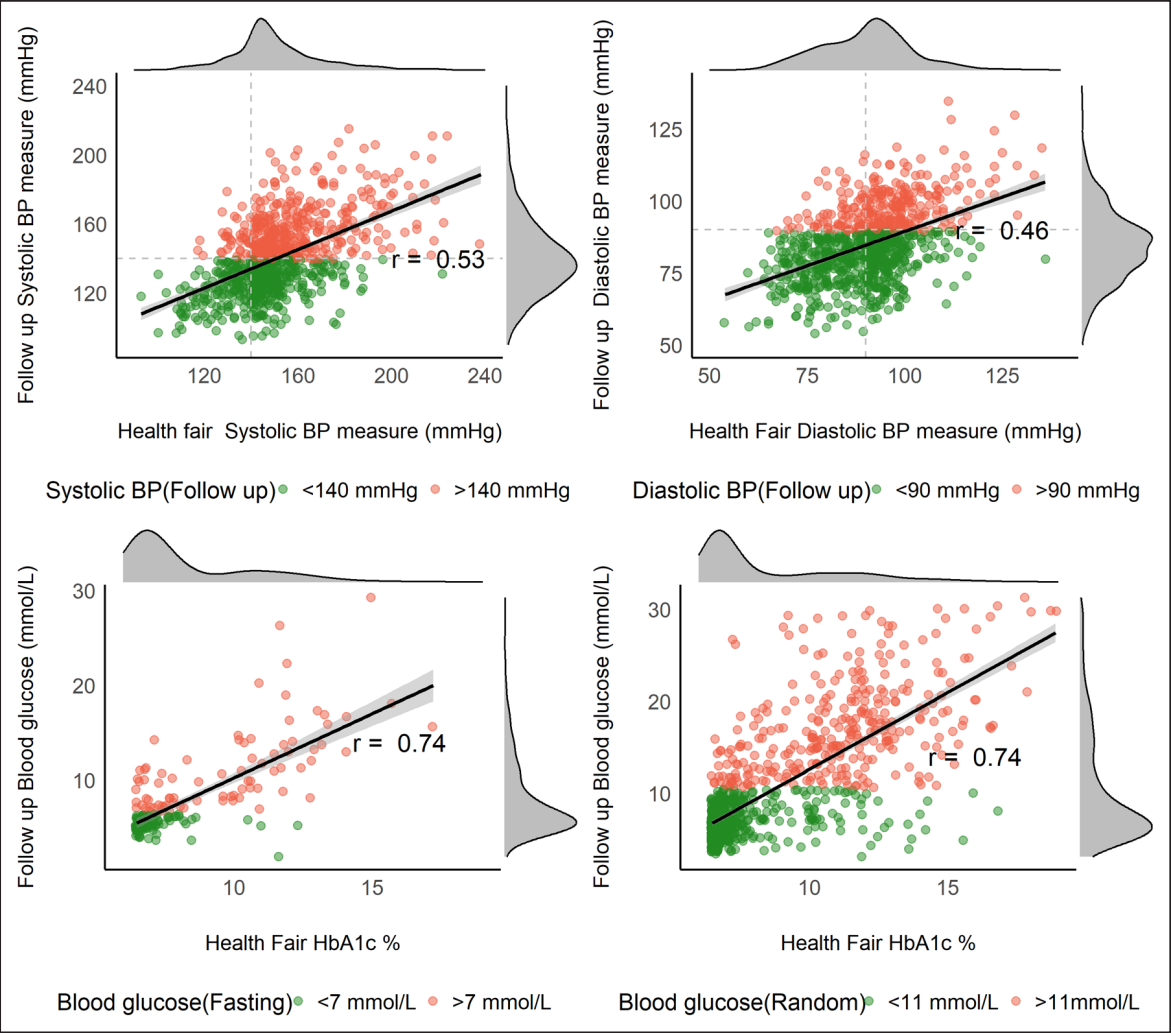
Distribution and correlation of blood pressure and HbA1c with blood glucose measures done at initial screening and confirmatory follow-up visit.

**Figure 3:**
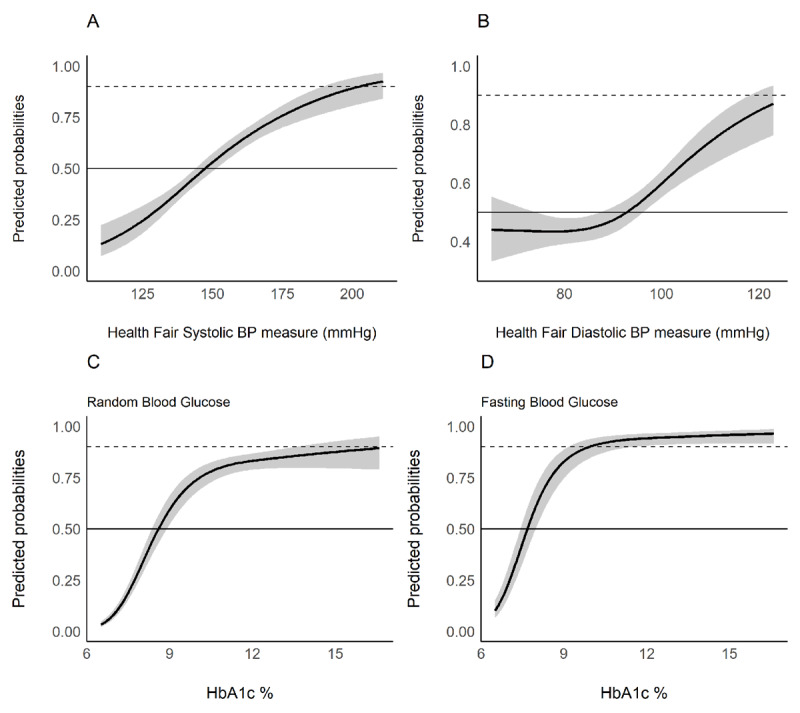
Predictions from a logistic regression model of the relationship between initial blood pressure and HbA1c glucose and the probability of having elevated blood pressure or blood glucose during the confirmatory follow-up visit.

We found a moderate correlation between initial and confirmatory BP screening (diastolic r = 0.46, P < 0.01; systolic BP r = 0.53, P < 0.01, Figure 2). However, of the 871 people who completed a home BP measurement by the nurse, only 451 (51.8%) had a repeated BP measurement meeting the criteria for raised BP. This corresponds to a positive predictive value of single screening versus two–day screening of 51.8% (95%CI 48.4–55.1). In a logistic regression model with elevated BP at the home visit as the outcome of interest, each 10mmHg increase in systolic BP above 140 and diastolic BP above 90 at the screening visit increased the probability of confirmatory BP above those thresholds by 8% (95%CI 6.5–10.0) and 4% (95%CI 2.0–6.6), respectively. However, to achieve a 90% positive predictive value to correctly identify elevated BP in two consecutive visits, the BP thresholds at the screening visit would be a systolic of 192 mm Hg and a diastolic of 123 mm Hg, respectively (Figure 3A–B). Using these thresholds would result in missing over 90% of those with confirmed elevated BP at the confirmatory measure.

### Diabetes Screening

There was a high correlation between HbA1c and the follow up blood glucose measures (fasting blood glucose r = 0.74, P < 0.01; random blood glucose r = 0.74, P < 0.01, Figure 2). However, only 395 (34.3%) of the 1,151 participants who completed home screening had either a fasting blood glucose of >7 mmol/L or a non-fasting blood glucose of >11 mmol/L. This corresponds to a positive predictive value of 34.3% (95%CI 31.6–37.2) when using a HbA1c threshold of 6.5%. When considering fasting and non-fasting blood glucose separately, the positive predictive values were 44.9% (95% CI 37.5–52.6) and 32.4% (95% CI 29.5–35.4), respectively. In logistic regression models, we found a 7% (95% CI 6.0–9.0) increased likelihood of a confirmed DM diagnosis with each 1% increase in HbA1c. To achieve a 90% positive predictive value to correctly identify participants with confirmed high blood glucose levels an HbA1c of 16.6% would be required (Figure 3C–D). However, this criterion misses over 95% of those confirmed as having elevated blood glucose during the confirmatory measure. In a sensitivity analysis excluding those with non-fasting blood glucose between 7.0–11.0 mmol/L, the positive predictive value increased from 34% to 45% (95 CI 41.9–48.9).

## Discussion

In a large community-based health fair program in South Africa, we found low positive predictive values of screening measurements of blood pressure and blood glucose to detect HTN and DM, compared to sequential testing on separate days. By instituting repeated BP and glucose measurements, we reduced potential clinical referrals by 48% for HTN and 66% for DM. For health systems capable of capacitating additional visits for confirmation (or exclusion) of DM and HTN, single-measure community-based programs might serve as an efficient prompt for initiating care engagement. However, in settings such as rural South Africa, where the public health system suffers from significant congestion and limited human resources for health, cross-sectional NCD screening programs run the risk of overburdening health services and transferring costs of over-diagnosis from the screening programs to the patient and public healthcare system.

The large and growing burden of HTN and DM in South Africa, along with the increasing appreciation that the majority of people with these conditions are unaware of their condition, requires urgent attention [5]. Among the most pressing of priorities is raising awareness of disease among those affected. In response to this realization, multiple prior programs have implemented health system referrals after community-based screenings for BP and DM [8,9,10,11,12,13,14]. Such programs have the benefit of bringing healthcare screenings out of congested primary healthcare clinics and into communities and individuals who might otherwise rarely interface with the formal health sector. Quasi-experimental studies following HTN screening programs in China and South Africa appear to show a benefit in terms of blood pressure control on a population level [12,22]. However, such screening programs also have potential drawbacks. Low rates of linkage and retention in care after large community-based screening programs have been reported in Malawi and South Africa [14,23]. Similar findings were reported in the United States in a meta-analysis of HTN screening programs [24]. In contrast, a screening program in Uganda that also included a travel voucher for those referred documented much higher rates of initial linkage [25]. Moreover, the public health care system, and particularly the NCD care program in South Africa has been shown to suffer from over-burdened clinics, long wait lines, and under-resourced clinics. Screening programs which result in large referrals (approximately 50% based on our findings) of individuals who do not meet criteria for treatment initiation runs the risk of burdening these clinics further and exposing individuals to nosocomial transmitted diseases.

Creative solutions that promote screening and increasing awareness of NCDs, but also prevent over-burdening of healthcare systems are needed. In our program, we provided a follow-up home visit prior to referring into the health system. Although this is likely to increase the accuracy of referral, we have yet to learn whether it resulted in improved retention in care. The additional costs and time required for second visits to either the patient or the healthcare system must also be considered. Other programs, such as the ComHIP program in Ghana, are currently evaluating community-based approaches to HTN care, that extends out of clinic services to include follow-up measurements and provision of medicines [10]. Effectiveness results of that program are forthcoming. Studies from southeast Asia and the United States have also shown promise of non-traditional NCD treatment through nursing led community-based care and provision of care at male-oriented venues [26,27].

We also sought to investigate whether more restrictive thresholds for referring to HTN and DM after an initial screen would be beneficial. However, we found that extremely high thresholds for systolic BP (192mm Hg), diastolic BP (123 mm Hg) and HbA1c (16.6%) were required to ensure that 90% of those referred would have confirmatory repeat measure on a second day. At these thresholds, we would have missed approximately 92% of HTN and 97% of DM cases who met criteria for treatment after sequential screening. Thus, our results do not suggest that there is a single higher screening threshold that would improve the positive predictive value of community-based screening without excluding a large proportion of people who do potentially require care. Prior studies have also shown that concomitant HTN screening along with HIV testing may lead to overdiagnosis of elevated BP due to transient elevations around the time of the HIV testing [28]. Consequently, in keeping with most international guidelines and the South African Primary Care Guidelines, two subsequent measurements on different days (excluding those with very high blood pressure or glucose measurement on first presentation) is required to help ensure accurate diagnosis of these conditions [20].

Our study should be interpreted in the context of a number of limitations. This screening program was conducted recently, so we do not yet have data on the resulting linkage to and retention care which resulted from the addition of a nurse home visit to confirm diagnosis and initiate the referral. Due to the nature of the study, we were not able to assess the number of participants who may have been false negatives at the health fair as they were not followed up. Furthermore, using the second visit measure as confirmatory raises the possibility missing legitimate cases with elevated measures who had normal readings at the follow-up leading under detection of disease. Lacking a gold standard of diagnosis, the current study cannot directly assess the accuracy of a single measure versus two consecutive measures. Our study was not set up to collect health system resources and costs, but future studies should carefully evaluate the tradeoffs between mass NCD screening and referrals, with the additional costs associated with repeated measures prior to referral. The use of 90% PPV to estimate an optimal threshold was not justified by prior literature and a less stringent value could have been used, however we feel this does not affect the overall message that a single measurement should not be used to refer individuals to health care settings. Finally, our study used HbA1c as an initial screen, with a fasting blood glucose as a confirmation of DM to account for South African guidelines and practice, but only a proportion of the confirmatory tests were done in the fasting state. Nonetheless, our false positivity rate for an initial screen remained high when we excluded individuals with non-fasting measures in the range between 7 mmol/L and 11 mmol/L.

Single measurement screening for HTN and DM in South Africa resulted in approximately a 50% rate of potentially unnecessary or premature referrals into the health sector for these conditions. Careful consideration of the potential benefits and risks of such screening programs will be essential to ensure they achieve their intended goal of improving NCD awareness and control. Cost-effectiveness and tradeoffs of such strategies versus repeated measure testing and/or community-based delivery of NCD care should be evaluated to further optimize care in such settings.

## Additional Files

The additional files for this article can be found as follows:

10.5334/gh.1083.s1 Supplementary Table 1. Demographic characteristics of complete home visits compared to LTFU.

10.5334/gh.1083.s2 Supplementary Table 2. Vukuzazi Study Team.
