# Supplementary material for: Pitfalls of Single Measurement Screening for Diabetes and Hypertension in Community-Based Settings
Source: Glob Heart. 2021 Dec 3;16(1):79. doi: 10.5334/gh.1083 (PMC8641532; doi:10.5334/gh.1083)
Supplement: Supplementary Table 1. — Demographic characteristics of complete home visits compared to LTFU. [file gh-16-1-1083-s1.pdf]

Supplementary material

	Home visit completed	Lost to follow-up
Characteristic	N = 1,886[1]	N = 248[1]
**Age**	59 (49, 68)	51 (39, 64)
**Age categories**		
15–24	62 (3.3%)	19 (7.7%)
25–44	287 (15%)	67 (27%)
45–64	932 (49%)	104 (42%)
65+	605 (32%)	58 (23%)
**Sex**		
Male	439 (23%)	87 (35%)
Female	1,447 (77%)	161 (65%)
**BMI**	32 (27, 37)	30 (25, 36)
**Systolic BP**	135 (120, 148)	139 (123, 149)
**Diastolic BP**	80 (72, 91)	84 (74, 94)
**HbA1c %**	6.60 (5.80, 7.50)	6.50 (5.80, 7.10)

[1]Median (IQR); n (%).

Supplementary Table 1. Demographic characteristics of complete home visits compared to LTFU.
