# Supplementary material for: Pitfalls of Single Measurement Screening for Diabetes and Hypertension in Community-Based Settings
Source: Glob Heart. 2021 Dec 3;16(1):79. doi: 10.5334/gh.1083 (PMC8641532; doi:10.5334/gh.1083)
Supplement: Supplementary Table 2. — Vukuzazi Study Team. [file gh-16-1-1083-s2.pdf]

Acknowledgments

We thank the residents of the Africa Health Research Institute demographic surveillance area and their leaders for many years of continuous engagement in population health research. We are particularly grateful to those who engaged with Vukuzazi either through participation or through considering participation. We appreciate members of the Community Advisory Board for their crucial input throughout the lifecycle of the project. We additionally acknowledge the partnership of the local and provincial Department of Health in their support of this project. We thank all the members of the Vukuzazi Study Team (Table below) for their valuable contributions to this study. We dedicate this manuscript to the memory of Hlobisile Chonco.

Vukuzazi Team: Staff who significantly contributed to the implementation and conduct of Vukuzazi.

***** Denotes team members who were closely involved with the design, implementation and oversight of Vukuzazi.

Name	Role
*Deenan Pillay	Principal Investigator (2017-2019)
*Willem Hanekom	Principal Investigator (2019-present)
*Emily Wong	Co-Principal Investigator
*Mark Siedner	Co-Principal Investigator
*Olivier Koole	Co-Principal Investigator (2017-2019)
*Thumbi Ndung'u	Co-investigator
*Thandeka Khoza	Co-investigator (2019-present)
*Kobus Herbst	Co-investigator
*Kathy Baisley	Co-investigator
*Janet Seeley	Co-investigator
*Alison Grant	Co-investigator
*Resign Gunda	Programme Manager
*Ashmika Surujdeen	Study Coordinator
*Theresa Smit	Head: Diagnostic Research
*Dickman Gareta	Head: Research Data Management
*Day Munatsi	Head: Research Data Systems
*Ngcebo Mhlongo	Study Physician
*Sanah Bucibo	Lead Nurse
*Tshwaraganang Modise	Research Data Manager
*Stephen Olivier	Statistician
*Gregory Ording-Jespersen	Laboratory Data Supervisor
*Innocentia Mpofana	Diagnostic Laboratory Manager
*Jaco Dreyer	Senior Research Data Manager
*Siyabonga Nxumalo	Research Data Manager
*Khadija Khan	Biorepository Manager
*Zizile Sikhosana	Somkhele Laboratory Supervisor
*Sashen Moodley	Microbiology Laboratory Supervisor
*Hollis Shen	Head: Exploratory Research Division
Kennedy Nyamande	Pulmonology Consultant
Mosa Suleman	Pulmonology Consultant
Jaikrishna Kalideen	Radiologist

Ramesh Jackpersad	Radiologist
Kgaugelo Moropane	Radiographer
Boitsholo Mfolo	Radiographer
Khabonina Malomane	Radiographer
Hlolisile Khumalo	Nursing Manager
Nompilo Buthelezi	Training Coordinator
Nozipho Mbonambi	Professional Nurse
Hloniphile Ngubane	Professional Nurse
Thokozani Simelane	Professional Nurse
Khanyisani Buthelezi	Professional Nurse
Sphiwe Ntuli	Professional Nurse
Nombuyiselo Zondi	Professional Nurse
Siboniso Nene	Professional Nurse
Bongumenzi Ndlovu	Enrolled Nurse
Talente Ntimbane	Enrolled Nurse
Mbali Mbuyisa	Enrolled Nurse
Xolani Mkhize	Enrolled Nurse
Melusi Sibiya	Enrolled Nurse
Ntombiyenkosi Ntombela	Enrolled Nurse
Mandisi Dlamini	Enrolled Nurse
Hlobisile Chonco	Enrolled Nurse
Hlengiwe Dlamini	Enrolled Nurse
Doctar Mlambo	Enrolled Nurse
Nonhlanhla Mzimela	Enrolled Nurse
Zinhle Buthelezi	Enrolled Nurse
Zinhle Mthembu	Enrolled Nurse
Thokozani Bhengu	Enrolled Nurse
Sandile Mthembu	Enrolled Nurse
Phumelele Mthethwa	Enrolled Nurse
Zamashandu Mbatha	Enrolled Nurse
Welcome Petros Mthembu	Enrolled Nurse
Anele Mkhwanazi	Clinical Research Assistant Supervisor
Mandlakayise Zikhali	Clinical Research Assistant Supervisor
Phakamani Mkhwanazi	Clinical Research Assistant
Ntombiyenhlanhla Mkhwanazi	Clinical Research Assistant
Rose Myeni	Clinical Research Assistant
Fezeka Mfeka	Clinical Research Assistant
Hlobisile Gumede	Clinical Research Assistant
Nonceba Mfeka	Clinical Research Assistant
Ayanda Zungu	Clinical Research Assistant
Hlobisile Gumede	Clinical Research Assistant
Nonhlanhla Mfekayi	Clinical Research Assistant
Smangaliso Zulu	Clinical Research Assistant
Mzamo Buthelezi	Clinical Research Assistant
Senzeni Mkhwanazi	Clinical Research Assistant
Mlungisi Dube	Clinical Research Assistant
Philippa Mathews	Clinical Governance
Siphephelo Dlamini	AHRI Nursing Manager

Hosea Kambonde	IT Systems Developer
Lindani Mthembu	Information Technology Assistant
Seneme Mchunu	Information Technology Assistant
Sibahle Gumbi	Research Admin Assistant
Tumi Madolo	Research Data Manager
Thengokwakhe Nkosi	Driver
Sibusiso Mkhwanazi	Driver
Sibusiso Nsibande	Driver
Mpumelelo Steto	Driver
Sibusiso Mhlongo	Driver
Velile Vellem	Driver
Pfarelo Tshivase	Driver
Jabu Kwinda	Driver
Bongani Magwaza	General Worker
Siyabonga Nsibande	General Worker
Skhumbuzo Mthombeni	General Worker
Sphiwe Clement Mthembu	General Worker
Antony Rapulana	Laboratory Technologist
Jade Cousins	Laboratory Technologist
Thabile Zondi	Laboratory Technologist
Nagavelli Padayachi	Laboratory Technologist
Freddy Mabetlela	Laboratory Technologist
Simphiwe Ntshangase	Laboratory Technician/LIMS Administrator
Nomfundo Luthuli	Laboratory Technician
Sithembile Ngcobo	Laboratory Technologist
Kayleen Brien	Laboratory Technologist
Sizwe Ndlela	Laboratory Technician
Nomfundo Ngema	Laboratory Technician
Nokukhanya Ntshakala	Laboratory Technician
Anupa Singh	Laboratory Technician
Rochelle Singh	Laboratory Technician
Logan Pillay	Laboratory Technician
Kandaseelan Chetty	Laboratory Technician
Ashentha Govender	Laboratory Technician
Pamela Ramkalawon	Laboratory Research Technician
Nondumiso Mabaso	Laboratory Intern
Kimeshree Perumal	Laboratory Intern
Senamile Makhari	Biorepository Laboratory Technician
Nondumiso Khuluse	Biorepository Laboratory Technician
Nondumiso Zitha	Biorepository Research Assistant
Hlengiwe Khathi	Biorepository Research Assistant
Mbuti Mofokeng	Clinical Specimen Driver/Laboratory Assistant
Nomathamsanqa Majozi	Public Engagement
Nceba Gqaleni	Public Engagement
Hannah Keal	Communications
Phumla Ngcobo	Communications

Costa Criticos	Operational Oversight
Raynold Zondo	Operational Oversight
Dilip Kalyan	Operational Oversight
Clive Mavimbela	Operational Oversight
Anand Ramnanan	Procurement
Sashin Harilall	Grants Office
